# Linking Brain Morphometry to Psychometric Measures and Energy‐Metabolic Biomarkers in Adults With Autism Spectrum Disorder

**DOI:** 10.1002/aur.70288

**Published:** 2026-06-05

**Authors:** Eleonora Esposto, Kathrin Nickel, Dominique Endres, Kimon Runge, Katharina Domschke, Thomas Lange, Marco Reisert, Anke Schumann, Cinzia Niolu, Ludger Tebartz van Elst, Simon Maier

**Affiliations:** ^1^ Department of Psychiatry and Psychotherapy, Medical Center—University of Freiburg, Faculty of Medicine University of Freiburg Freiburg Germany; ^2^ Department of Systems Medicine Tor Vergata University of Rome Rome Italy; ^3^ German Center for Mental Health (DZPG) Partner Site Berlin/Potsdam Berlin Germany; ^4^ Division of Medical Physics, Department of Diagnostic and Interventional Radiology, Medical Center—University of Freiburg, Faculty of Medicine University of Freiburg Freiburg Germany; ^5^ Department of Stereotactic and Functional Neurosurgery, Faculty of Medicine, Medical Center—University of Freiburg University of Freiburg Freiburg Germany; ^6^ Center for Children's and Women's Medicine, Center for Pediatrics, Department of General Pediatrics, Adolescent Medicine and Neonatology, Medical Center—University of Freiburg/Faculty of Medicine University of Freiburg Freiburg Germany; ^7^ Psychiatry and Clinical Psychology Unit Fondazione Policlinico Tor Vergata Rome Italy

**Keywords:** acylcarnitines, autism spectrum disorder, brain morphometry, cortical thickness, magnetic resonance spectroscopy, mitochondrial energy biomarkers

## Abstract

Autism spectrum disorder (ASD) is associated with differences in neurodevelopment and altered metabolism, yet the interplay between brain morphometry, mitochondrial and energy metabolism biomarkers, and autistic traits in adults remains poorly understood. This study investigates the link between brain structure, psychometric measures, and both central and peripheral metabolic biomarkers in adults with ASD. We studied 145 adults, including 74 with ASD and 71 control participants (CON) using high‐resolution 3‐Tesla MRI to assess cortical thickness, subcortical and global brain volumes. Central energy metabolism was indexed by the posterior‐cingulate lactate + threonine (Lac^+^) peak quantified with proton‐MRS. We examined associations between biomarkers of mitochondrial function and energy metabolism (including lactate, pyruvate, creatine kinase, and multiple acylcarnitines). Psychometric evaluations included measures of ASD and attention‐deficit/hyperactivity disorder (ADHD) symptom severity, as well as other psychiatric comorbidities. Between‐group differences and correlations were assessed using robust statistics, controlling for age, sex, image quality, and total intracranial volume. Adults with ASD showed significantly larger bilateral caudate volumes compared to CON. Within the ASD group, higher ADHD symptom severity in childhood correlated with reduced cortical thickness in multiple frontal and temporal regions. Among metabolic markers, acylcarnitine C5:1 was positively associated with right insular cortex thickness, while C18:1‐OH and C18:2 levels correlated positively with caudate volume. Caudate nucleus volume is associated not only with an ASD diagnosis but also with specific peripheral energy‐metabolism blood markers, such as specific acylcarnitines. Alterations in cortical thickness were also correlated with acylcarnitine levels and, to a greater extent, with co‐occurring ADHD symptoms. While alterations in cortical thickness and basal ganglia structure have been previously described in ASD and comorbid ADHD, the linkage between mitochondrial and energy metabolism biomarkers with neuroanatomical alterations in ASD is, to our knowledge, a novel observation that warrants further investigation.

## Summary

Autism spectrum disorder (ASD) is known to affect brain development and the way the brain uses energy. However, how brain structure, energy metabolism, and autistic traits are connected in adults is still unclear. In this study, we examined brain scans and blood samples from 145 adults, some with ASD and some without (control participants). We also looked at their psychological traits, including symptoms of ADHD at childhood. We found that adults with ASD had a larger part of the brain called the caudate nucleus. In those with stronger ADHD symptoms during childhood, frontal and lateral brain regions were thinner. We also discovered that specific molecules in the blood related to energy use, called acylcarnitines, were linked to differences in brain structure. Some of these markers were connected to the size of the caudate nucleus and the thickness of the insular cortex. These results suggest that changes in brain structure in adults with ASD may be related not only to behavior and symptoms, but also to differences in how the brain produce and use energy. This connection between metabolism and brain structure could help us better understand autism and related conditions in the future.

## 1 Introduction

Autism spectrum disorder (ASD) is a complex neurodevelopmental condition characterized by deficits in social communication and repetitive behaviors, with substantial heterogeneity in clinical presentation. Structural neuroimaging studies have identified differences in global and regional brain measures in individuals with ASD, including white matter volume, cortical thickness (CT), and subcortical volume (SV), with some findings linking these alterations to specific symptom domains (Sears et al. 1999; Herbert et al. 2003; Hollander et al. 2005; Ecker et al. 2022). However, findings have been inconsistent, particularly, in adulthood, with some studies suggesting attenuation of structural differences over time, while others report persistent brain overgrowth across development (Libero et al. 2016; van Rooij et al. 2018). But it remains unclear to what extent brain morphometric differences in ASD primarily reflect core autism traits or are influenced by co‐occurring conditions.

Growing evidence suggests that metabolic alterations may contribute to ASD‐related brain changes and behavioral symptoms (Bauman and Kemper 2005; Lord et al. 2020). Energy‐metabolism deficiencies, oxidative stress, and disruptions in amino acid metabolism may be implicated in its pathophysiology (Rossignol and Frye 2012). Meta‐analytic work estimates that up to 5% of individuals with ASD meet criteria for primary mitochondrial disease versus about 0.01% in the general population, and that 30%–80% of affected children display at least one biomarker of mitochondrial dysfunction (Rossignol and Frye 2012; Rose et al. 2018; Frye et al. 2019). Direct markers such as elevated blood lactate and pyruvate (Giulivi et al. 2010; Oh et al. 2020) and altered acylcarnitine profiles (Nickel et al. 2023; Frye et al. 2024) have been consistently reported in ASD cohorts.

Carnitine metabolism in particular, central to fatty‐acid transport into mitochondria, has attracted interest not only for its role in energy production and neuroprotection (Aslan et al. 2021; Alhasaniah 2023) but also because aberrant acylcarnitine patterns have been linked to regional brain atrophy and cognitive decline in Alzheimer's disease (Ciavardelli et al. 2016). Similarly, elevated brain lactate, a marker of impaired oxidative metabolism, has been observed in bipolar depression (Machado‐Vieira et al. 2017), Parkinson's dementia (Bowen et al. 1995), and schizophrenia (Rowland et al. 2016). Despite this cross‐disorder evidence that bioenergetic markers can covary with gray‐matter volume and CT, the relationship between systemic and brain‐derived energy‐metabolism biomarkers and brain morphometry remains poorly delineated in ASD, especially in adult cohorts.

This study aims to investigate the correlation between systemic energy‐metabolic markers, including blood‐derived biomarkers and carnitine metabolites, a central marker of cerebral energy metabolism (posterior cingulate lactate + threonine, Lac^+^, obtained via proton magnetic resonance spectroscopy [MRS]), psychometric measures, including autism symptom severity, and co‐occurring attention‐deficit/hyperactivity disorder (ADHD) symptoms, and brain morphometry in adults with ASD. Specifically, we examine differences in global brain volumes (GVs), CT, and SV between adults with ASD and control participants (CONs) and assess how psychometric and metabolic factors relate to these structural measures.

We hypothesize that there will be regional but no global differences between the ASD and CON groups (Haar et al. 2016; van Rooij et al. 2018), particularly in regions such as the medial prefrontal cortex, the anterior cingulate cortex, and the superior temporal gyrus, implicated in social cognition, communication, and emotional regulation (Zahn et al. 2007; Bzdok et al. 2013; Apps et al. 2016). Additionally, we expect alterations in SV, in particular the caudate nucleus, a region potentially linked to repetitive and stereotyped behaviors (Sears et al. 1999; Hollander et al. 2005), as well as in the amygdala and hippocampus, considering their role in social and affective processing (Schumann et al. 2004; Nacewicz et al. 2006).

Based on previous studies examining the relationship between brain morphometry and psychometric measures in ASD, we expect autism‐specific traits to correlate with CT in regions involved in social cognition (Nunes et al. 2020; Nees et al. 2022). Given prior research suggesting mitochondrial dysfunction and altered lipid metabolism in ASD (Frye et al. 2019; Aslan et al. 2021), we also expect brain morphometry to be associated with peripheral blood markers, including acylcarnitines, as well as with the central MRS‐derived Lac^+^ signal. To our knowledge, this is the first study to integrate peripheral and central metabolic indices with brain morphometry in an adult ASD cohort.

## 2 Methods

Certain aspects of this cohort have been reported previously, specifically, cerebral lactate levels measured by MRS (Maier et al. 2023) and peripheral metabolic blood markers (Nickel et al. 2023). The present study now focuses on brain morphometry in relation to ASD, these blood markers, and cerebral lactate levels.

### 2.1 Ethics

The study was conducted at the Department of Psychiatry and Psychotherapy of the University of Freiburg. The research received ethical approval from the Ethics Committee of the Medical Center—University of Freiburg (Approval no. EK‐Freiburg: 209/18), in accordance with the Declaration of Helsinki. All participants provided written informed consent prior to inclusion in the study.

### 2.2 Diagnostic and Eligibility Criteria

Adults aged 18–65 years were eligible for inclusion. Individuals with ASD were recruited from current and former patients of the Department of Psychiatry and Psychotherapy at the University Medical Center Freiburg. Furthermore, patients were only included if they received a diagnosis of ASD according to the *Diagnostic and Statistical Manual of Mental Disorders* (DSM‐5; 299.00) and International Classification of Disease (ICD‐10; F84.5), with confirmation through the Autism Diagnostic Observation Schedule (ADOS) (Lord et al. 1989) and Autism Diagnostic Interview Revised (ADI‐R) (Lord et al. 1994) for cases with diagnostic ambiguity. Participants with secondary genetic causes of ASD were excluded to maintain a clinically homogeneous sample. Genetic markers, including physical indicators like facial dysmorphisms and medical histories suggestive of genetic syndromes (e.g., cardiac or seizure conditions), were evaluated to screen out such cases.

The CON were recruited as community volunteers through internal hospital announcements at the University Medical Center Freiburg and were not recruited as patients. All participants (ASD and CON) underwent the same diagnostic and symptoms assessment battery, including the Structured Clinical Interview for DSM‐IV Axis I and II disorders (SCID I and II) (First and Gibbon 2004), and the Symptom Checklist‐90‐revised (SCL‐90‐R) (Derogatis and Savitz 1999). In controls, these instruments were used to exclude current or lifetime psychiatric disorders; in the ASD group, they were used to characterize comorbidity. Depressive symptoms and retrospective childhood ADHD symptoms were assessed in both groups using the Beck Depression Inventory‐II (BDI‐II) (Strunk and Lane 2017) and the Wender Utah Rating Scale (WURS‐k) (Retz‐Junginger et al. 2002), respectively. To assess autistic symptoms in the ASD group and rule them out in the CON group, the Autism Spectrum Quotient (AQ) (Baron‐Cohen et al. 2001), the Empathy Quotient (EQ) (Baron‐Cohen and Wheelwright 2004), and the Social Responsiveness Scale‐2 (SRS‐2) (Bruni 2014) were employed.

All participants were screened for general magnetic resonance imaging (MRI) contraindications (e.g., metallic implants or claustrophobia), neurological or metabolic conditions (e.g., seizure disorders or diabetes), obesity (body mass index > 30.0 kg/m^2^), and regular benzodiazepine use. In the ASD group, individuals with a history of bipolar disorder, psychosis, or substance abuse were excluded, whereas in the CON group, any history of psychiatric illness led to exclusion. Psychotropic medication use was documented in the ASD group but did not preclude study participation. Participants were instructed to refrain from physical exercise 24 h before data acquisition.

### 2.3 Further Psychometric Assessments

The assessment protocol included: the Multiple‐Choice Vocabulary Test (MWT‐B) for crystallized intelligence (IQ) (Wittorf et al. 2014) and the State–Trait Anxiety Inventory (STAI) for symptoms of anxiety (Spielberger 2012).

### 2.4 Blood Metabolites Acquisition and Analysis

Blood samples were collected and analyzed according to previously published protocols (Nickel et al. 2023), including metabolite quantification and quality control. Measured parameters included the liver enzymes alanine aminotransferase and aspartate aminotransferase, energy‐metabolic markers such as lactate, pyruvate, lactate/pyruvate ratio, creatine kinase, carnitine metabolites (total and free carnitine), and a panel of acylcarnitines, reflecting fatty‐acid oxidation and mitochondrial β‐oxidation flux.

### 2.5 MRI Acquisition

MRI scans were conducted on a Siemens 3 Tesla Magnetom Prisma system (Siemens Healthineers, Munich, Germany) at the Imaging Center of the Department of Radiology of the Medical Center—University of Freiburg. A 20‐channel head coil was used for optimal signal reception. An MPRAGE sequence was employed to obtain high‐resolution anatomical images with the following parameters: field of view = 256 × 256 × 160 mm^3^, voxel size = 1 × 1 × 1 mm^3^, repetition time (TR) = 2000 ms, echo time (TE) = 4.11 ms, and flip angle = 12°.

### 2.6 MRS Acquisition

Lactate levels in the posterior cingulate cortex were measured with a MEGA‐semiLASER MRS sequence (Maier et al. 2023) with the following parameters: TR = 1650 ms, TE = 142 ms, flip angle = 90°, 192 averages, voxel centered at 2.0 ppm. For editing, a pulse with a 60 Hz full width at half maximum (FWHM) was applied at 4.1 ppm during edit‐ON and at 5.3 ppm during edit‐OFF. Absolute water was quantified by repeating the protocol without water suppression (voxel centered at 4.7 ppm, 16 averages). The 25 × 25 × 25 mm^3^ single‐voxel MRS was placed over the splenium near the tentorium cerebelli in the occipital region of the corpus callosum, optimizing the signal‐to‐noise ratio for the posterior cingulate cortex while minimizing cerebrospinal fluid (CSF) inclusion.

### 2.7 Data Preprocessing

For the extraction of GV measures (gray matter volume, total white matter volume, CSF volume, total intracranial volume [TIV], cerebellar volume) and CT estimates, CAT12 (Version: CAT12.9 r2517) running in MATLAB (R2021a) with default parameters (http://dbm.neuro.uni‐jena.de/cat/) was used to automatically preprocess T1‐weighted MR images (Gaser et al. 2024). This well‐established computational framework employs standard segmentation techniques to differentiate brain tissues and provides reliable structural imaging data for subsequent analysis. For the ROI‐wise CT estimates the mean CT estimates for each of the 80 ROIs (40 per hemisphere) of the DKT 40 atlas was calculated. To automatically segment SV information, we used Free Surfer version 7.2.0 (https://www.freesurfer.net/fswiki/FreeSurferWiki), running the recon‐all command with default parameters (Fischl 2012). For further analysis, we proceeded with the following SV: Left Lateral Ventricle; Right Lateral Ventricle; Left Thalamus; Right Thalamus; Left Caudate Nucleus; Right Caudate Nucleus; Left Putamen; Right Putamen; Left Globus Pallidus; Right Globus Pallidus; Left Hippocampus; Right Hippocampus; Left Amygdala; Right Amygdala; Left Nucleus Accumbens; Right Nucleus Accumbens. All statistical analyses were performed using R (Version 4.3.3) running on RStudio (Version 2024.09.0 + 375; Posit Software, Boston, MA, USA). All global volumes except TIV were divided by TIV to adjust for individual differences in TIV, hence, reporting relative GV. For details on lactate quantification from the MRS spectra with Osprey (v2.5.0), please refer to (Maier et al. 2023). Since lactate and threonine resonate around 1.31 ppm, their signals significantly overlap, making it difficult to distinguish them separately. Therefore, we report their combined signal as Lac+.

### 2.8 Potential Nuisance Covariates

Potential nuisance covariates, including age, age^2^, sex, body mass index, TIV, and image quality rating (IQR; derived from CAT12), were screened using the Boruta package for R (Kursa and Rudnicki 2010). Boruta is an all‐relevant feature‐selection method that compares the importance of observed variables with that of randomly permuted shadow variables and thereby identifies variables that are informative for the outcome beyond random noise. In this step, age, sex, and IQR were identified as relevant covariates for global volume measures, whereas for various cortical and subcortical ROIs, TIV was additionally identified as relevant in the Boruta screening. To account for the potentially confounding effects of these variables, we employed a robust linear regression model using the lmrob function of the robustbase package (Finger 2010) in R in combination with the package's predict function. For each cortical, subcortical, and global ROI (Theußl et al. 2020), the ROI measure was included as the dependent variable, while age, sex, IQR, and in the case of SV and CT, TIV were included as independent variables. All further statistics were performed with volume and thickness measures adjusted for the influence of these potentially confounding variables.

### 2.9 Between‐Group Comparison and Correlations

Due to deviations from normal distribution, robust Yuen's *t*‐tests from the WRS package were used to test for between‐group comparisons (Mair and Wilcox 2020) and Spearman's correlations to test for relation between the volume and thickness measures with psychometric, blood‐, and MRS‐derived parameters. Separately, for each modality (CT, SV, and global volume measures) and each predictor variable (group, psychometric score, blood‐derived measure, MRS‐derived measure) we controlled for multiple testing across the different ROIs using false‐discovery rates (FDR) according to Benjamini and Hochberg (Benjamini et al. 2001). All tests were two‐tailed; significance was set at *p‐*values < 0.05, and effect sizes were reported as Cohen's *d*.

### 2.10 Influence of Medication

To assess the potential confounding effect of medication, we repeated the adjustment for confounding effects using the robustbase package (Finger 2010) incorporating the package's “predict” function described in the “Confounding effects” section. In these models we included one of the following medication classes as an additional covariate: (1) stimulants, (2) antidepressants, and (3) antipsychotics. We report all tests that remained significant in the initial group comparisons and correlational analyses after adjustment using the medication‐adjusted volume and thickness measures.

### 2.11 Sensitivity Analysis

As a sensitivity analysis, extreme values were identified objectively using modified *z*‐scores based on the median absolute deviation (MAD), calculated separately within diagnostic group for the variables shown in each scatterplot; observations with |modified *z*| > 3.5 on either axis were excluded from the corresponding supplementary extreme‐value‐deprived analyses (Figures S2–S4).

## 3 Results

### 3.1 Sample Characteristics

The sample included 145 participants (74 ASD and 71 CON). Mean age in the ASD group was 36.0 ± 12.1 years and in the CON group 31.8 ± 9.9 years (*p* = 0.022). No differences were observed in sex, body mass index, or IQ. Psychometric assessments revealed higher autism‐related traits (SRS‐2, AQ, and EQ), ADHD symptoms in childhood (WURS‐k), and depressive symptoms (BDI‐II) in the ASD group compared to CON (all *p* < 0.001). Psychotropic medication use was only reported in the ASD group. During MRI‐debriefing, no participants reported experiencing anxiety or panic during scanning. The image quality assessment performed with CAT12 indicated good overall image quality for all subjects. Additionally, quality metrics, including noise/contrast ratio, surface defect number, and surface defect area, were comparable across groups. The results are summarized in Table 1; additional details are provided in Table S1.

**TABLE 1:** Demographic, psychometric, and medication data for ASD and CON groups.

Characteristic	ASD, *N* = 74	CON, *N* = 71	*p*
*Demographic characteristics*			
Age, years, mean (SD)	36.0 (12.1)	31.8 (9.9)	0.022
Female, no. (%)	26 (35)	27 (38)	0.70
Male, no. (%)	48 (65)	44 (62)	
Body mass index, kg/m^2^, mean (SD)	25.0 (5.1)	23.7 (5.7)	0.13

Cognitive function	Mean (SD)	Mean (SD)	
IQ (MWT‐B)	114.6 (14.8)	114.6 (13.9)	> 0.90

Psychometric evaluations	Mean (SD)	Mean (SD)	
Autism Spectrum Quotient (AQ)	35.6 (8.1)	12.1 (5.7)	< 0.001
Empathy Quotient (EQ)	22.0 (11.1)	47.5 (10.6)	< 0.001
Social Responsiveness Scale‐2 (SRS‐2)	108.2 (28.8)	34.7 (14.4)	< 0.001
Wender Utah Rating Scale (WURS‐k)	27.6 (15.9)	12.7 (10.0)	< 0.001
Beck Depression Inventory‐II (BDI‐II)	15.7 (13.1)	3.8 (4.6)	< 0.001

Medication	No. (%)	No. (%)	
Typical antipsychotics	2 (2.7)	0	
Atypical antipsychotics	7 (9.5)	0	
SSRIs/SNRIs	16 (22)	0	
Other antidepressants	16 (22)	0	
Lithium	2 (2.7)	0	
Psychostimulants	4 (5.4)	0	
Anticonvulsants	1 (1.4)	0	
Benzodiazepines (occasionally)	2 (2.7)	0	
Oral contraceptives	3 (4.1)	5 (7.0)	0.50

*Note:* Demographic data between the ASD and CON groups showed a difference in age, as well as higher scores in the psychometric evaluations in individuals with ASD. None of the CON participants was taking psychiatric medications.

Abbreviations: AQ, Autism Spectrum Quotient; ASD, autism spectrum disorder; BDI‐II, Beck Depression Inventory‐II; CON, control participants; EQ, Empathy Quotient; IQ, crystallized intelligence; MWT‐B, Multiple‐Choice Vocabulary Test; SD, standard deviation; SNRI, serotonin–norepinephrine reuptake inhibitor; SRS‐2, Social Responsiveness Scale‐2; SSRI, selective serotonin reuptake inhibitor; WURS‐k, Wender Utah Rating Scale.

### 3.2 Between‐Group Comparisons in Brain Morphometry

No differences were observed in relative global volumes (Table 2).

**TABLE 2:** Comparison of global brain volume relative to TIV between the ASD and CON groups.

Characteristic	ASD, *N* = 74	CON, *N* = 71	*p*
	Mean (SD)	Mean (SD)	
GM volume/TIV	0.457 (0.018)	0.451 (0.018)	0.07
WM volume/TIV	0.356 (0.018)	0.359 (0.019)	0.69
CSF volume/TIV	0.186 (0.025)	0.188 (0.025)	0.66
Cerebellum volume/TIV	0.100 (0.008)	0.099 (0.006)	0.65
TIV	1562.7 (107.2)	1610.2 (121.0)	0.09

*Note:* After corrections, no differences were observed in global brain volumes between the two groups.

Abbreviations: ASD, autism spectrum disorder; CON, control participants; CSF, cerebrospinal fluid; GM, gray matter; *N*, number of participants; SD, standard deviation; TIV, total intracranial volume; WM, white matter.

**FIGURE 1:**
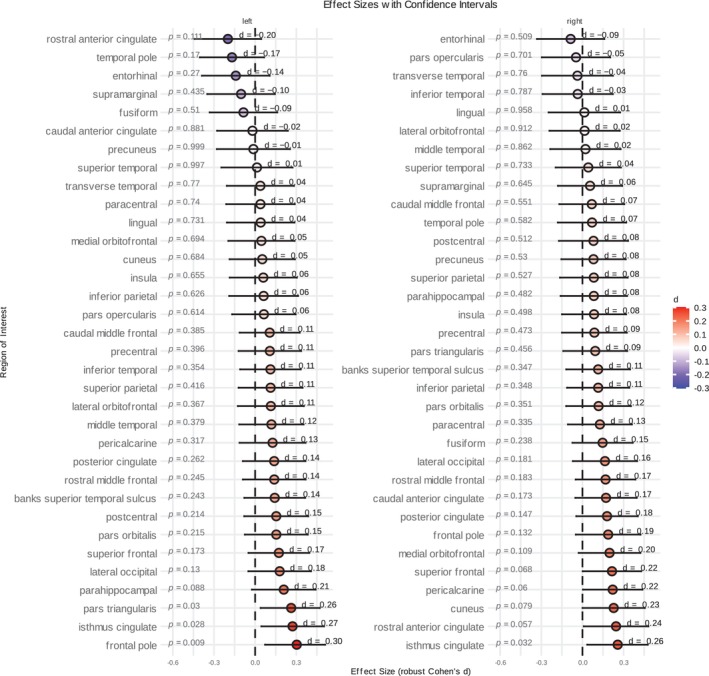
Cortical analysis (before correction for multiple testing) of various brain regions showing effect sizes and confidence intervals for group differences in cortical thickness between the ASD and CON groups. The ASD group showed significantly larger cortical thickness in the left frontal pole, pars triangularis, bilateral isthmus cingulate, and right rostral anterior cingulate cortex.

**FIGURE 2:**
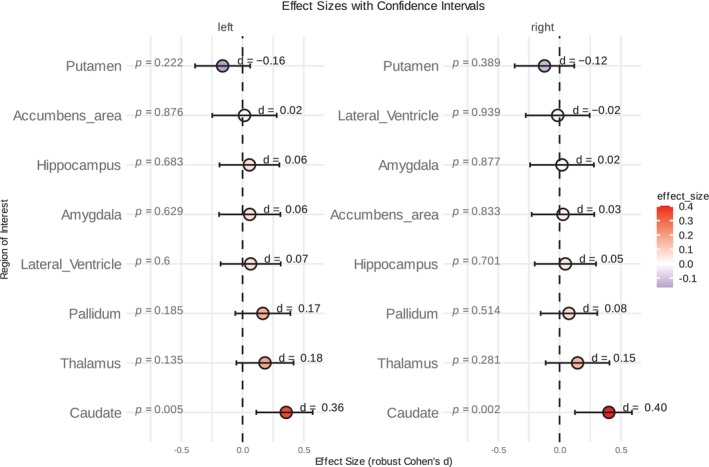
Subcortical analysis (before correction for multiple testing) of various brain regions showing effect sizes and confidence intervals for group differences in subcortical volume between the ASD and CON groups. The ASD group showed significantly larger volumes of both the left and right caudate nuclei.

Before correction for multiple testing, CT was significantly larger in the left frontal pole, pars triangularis, bilateral isthmus cingulate, and right rostral anterior cingulate cortex in the ASD group (Figure 1). Similarly, subcortical analysis before correction for multiple testing showed significantly larger volumes of the left and right caudate nuclei in the ASD group (Figure 2). After correction for multiple comparisons, only bilateral caudate volumes remained significantly larger in the ASD group (Table 3).

**TABLE 3:** Comparison of subcortical volumes between the ASD and CON groups.

Region	Hemisphere	*t*	Effect size	*p*	Adjusted *p*
Nucleus accumbens	Left	0.161	0.024	0.88	0.94
Nucleus accumbens	Right	0.215	0.028	0.83	0.94
Amygdala	Left	0.494	0.062	0.63	0.94
Amygdala	Right	0.146	0.02	0.88	0.94
Caudate nucleus	Left	2.868	0.358	0.005	**0.042**
Caudate nucleus	Right	3.213	0.404	0.002	**0.038**
Hippocampus	Left	0.41	0.057	0.68	0.93
Hippocampus	Right	0.374	0.051	0.70	0.93
Lateral ventricles	Left	0.52	0.068	0.60	0.93
Lateral ventricles	Right	−0.074	0.016	0.94	0.94
Globus pallidus	Left	1.322	0.171	0.19	0.71
Globus pallidus	Right	0.623	0.082	0.51	0.93
Putamen	Left	−1.229	0.16	0.22	0.71
Putamen	Right	−0.857	0.121	0.39	0.89
Thalamus	Left	1.45	0.183	0.14	0.71
Thalamus	Right	1.057	0.15	0.28	0.75

*Note:* Group comparisons of subcortical volumes between individuals with ASD and CON, stratified by hemisphere. Significant group differences were observed in the caudate nuclei, with both left (adjusted *p* = 0.042, *d* = 0.358) and right caudate (adjusted *p* = 0.038, *d* = 0.404) volumes significantly larger in the ASD group. No other subcortical structures showed significant volume differences after correction for multiple comparisons (adjusted *p* > 0.05). Effect sizes for these differences (Cohen's *d*) were in the small‐to‐moderate range.

Abbreviations: ASD, autism spectrum disorder; CON, control participants.

### 3.3 Psychometry and Brain Morphometry

**FIGURE 3:**
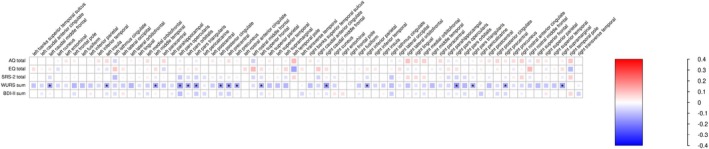
Correlation matrix of psychometric measures with cortical thickness across regions of interest in the ASD group. Box color indicates the direction of the correlation, with red indicating a positive correlation and blue indicating a negative correlation. Box size reflects the strength of the correlation, with larger boxes indicating stronger correlations. Significance after correction for multiple testing is indicated by an asterisk (*p* < 0.05). Abbreviations: AQ, Autism Spectrum Quotient; BDI‐II, Beck Depression Inventory‐II; EQ, Empathy Quotient; SRS‐2, Social Responsiveness Scale‐2; WURS‐k, Wender Utah Rating Scale.

No significant associations were found between psychometric traits and GVs. Within the ASD group, however, a significant negative correlation between ADHD symptoms in childhood (WURS‐k) and CT in left caudal middle frontal (*r* = −0.32, *p* = 0.007), left insula (*r* = −0.31, *p* = 0.011), left middle temporal (*r* = −0.32, *p* = 0.007), left pars opercularis (*r* = −0.4, *p* = 0.001), left pars orbitalis (*r* = −0.31, *p* = 0.009), left pars triangularis (*r* = −0.36, *p* = 0.002), left posterior cingulate (*r* = −0.35, *p* = 0.004), left precentral (*r* = −0.35, *p* = 0.003), left precuneus (*r* = −0.32, *p* = 0.008), left superior frontal (*r* = −0.31, *p* = 0.01), right caudal middle frontal (*r* = −0.38, *p* = 0.001), right inferior parietal (*r* = −0.3, *p* = 0.011), right pars opercularis (*r* = −0.39, *p* = 0.001), right pars triangularis (*r* = −0.3, *p* = 0.011), right precentral (*r* = −0.31, *p* = 0.009), and right supramarginal (*r* = −0.35, *p* = 0.003) cortices (Figure 3) after correction for multiple comparisons. When the same correlations were tested across the full cohort (ASD + CON combined), no associations between psychometric scores and global or regional morphometric measures remained significant after correction for multiple comparisons. There were no significant correlations between psychometric traits and SV after correction for multiple comparisons.

### 3.4 Metabolic Markers (Blood and MRS) and Brain Morphometry

**FIGURE 4:**
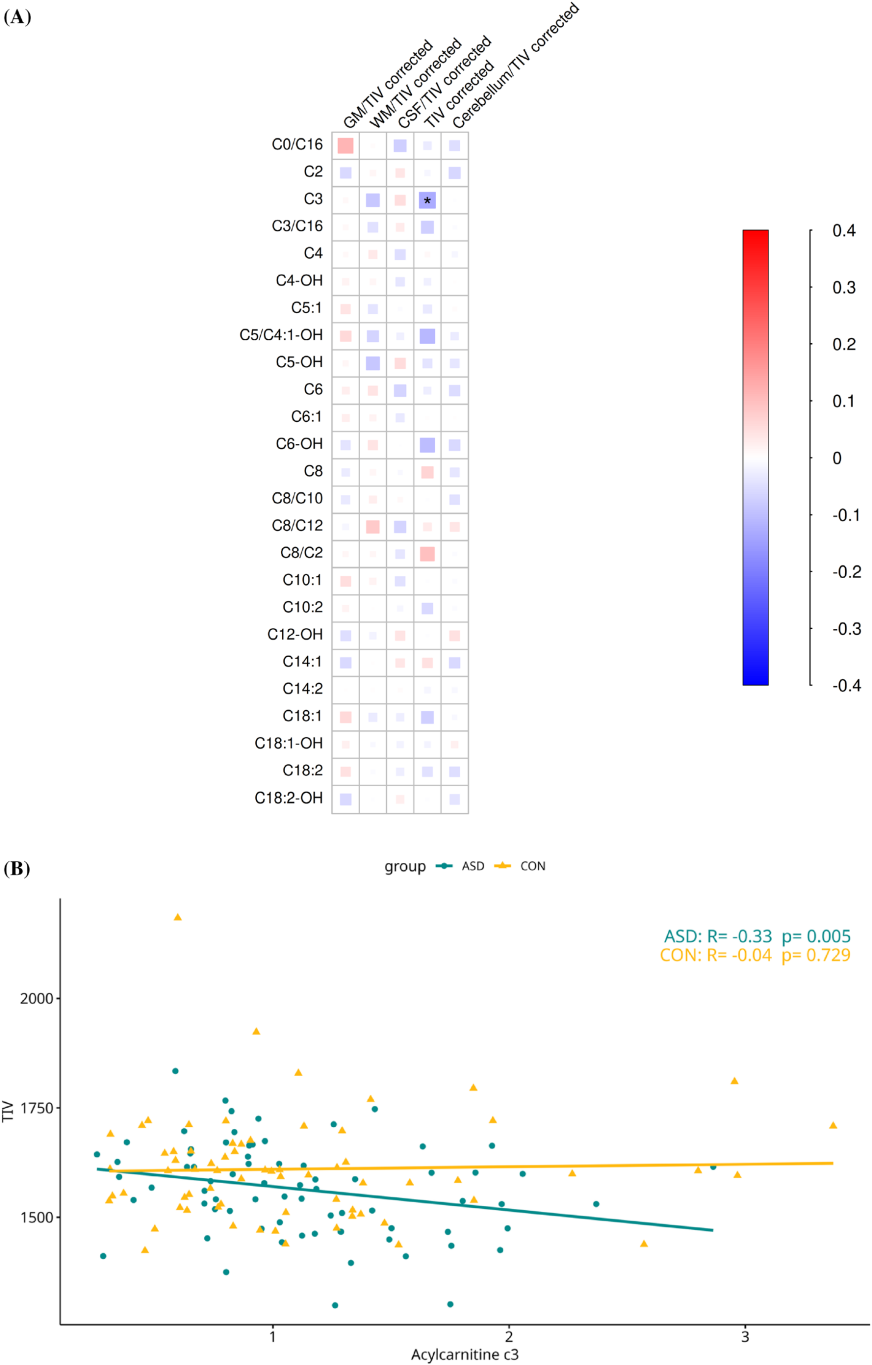
(A) Correlation matrix of acylcarnitine concentration data and global brain volume measures across regions of interest in the ASD group. Box color indicates the direction of the correlation, with red indicating a positive correlation and blue indicating a negative correlation. Box size reflects the strength of the correlation, with larger boxes indicating stronger correlations. Significance after correction for multiple testing is indicated by an asterisk (*p* < 0.05). Results showed that C3 was negatively correlated with TIV. (B) Scatterplot showing the association between propionylcarnitine (C3) levels and TIV in the ASD and CON groups. The yellow line represents the CON group, and the green line represents the ASD group. A significant negative correlation between C3 and TIV was observed within the ASD group after correction for multiple testing. Reported *p‐*values in the plot are shown before correction for multiple testing. Abbreviations: ASD, autism spectrum disorder; CON, control participants; CSF, cerebrospinal fluid; C0/C16, free carnitine/palmitoylcarnitine; C2, acetylcarnitine; C3, propionylcarnitine; C3/C16, propionylcarnitine/palmitoylcarnitine; C4, butyrylcarnitine; C4‐OH, hydroxy‐butyrylcarnitine; C5:1, tiglylcarnitine; C5 + C4:1‐OH, isovalerylcarnitine+3‐hydroxy‐butyrylcarnitine; C5‐OH, 3‐hydroxy‐isovalerylcarnitine; C6, hexanoylcarnitine; C6:1, hexenoylcarnitine; C6‐OH, 3‐hydroxy‐hexanoylcarnitine; C8, octanoylcarnitine; C8/C10, octanoylcarnitine/decanoylcarnitine; C8/C12, octanoylcarnitine/lauroylcarnitine; C10:1, decanoylcarnitine; C10:2, decadienoylcarnitine; C12‐OH, 3‐hydroxy‐dodecanoylcarnitine; C14:1, tetradecanoylcarnitine; C14:2, tetradecadienoylcarnitine; C18:1, octadecenoylcarnitine; C18:1‐OH, 3‐hydroxy‐oleylcarnitine; C18:2, octadecadienylcarnitine; C18:2‐OH, 3‐hydroxylinoleylcarnitine; GM, gray matter; TIV, total intracranial volume; WM, white matter.

No correlations were observed between peripheral blood markers and GVs, CT, or SV, except for acylcarnitines. A negative correlation between Propionylcarnitine (C3) and TIV (*r* = −0.33, *p* = 0.005) was found in the ASD group (Figure 4A,B).

**FIGURE 5:**
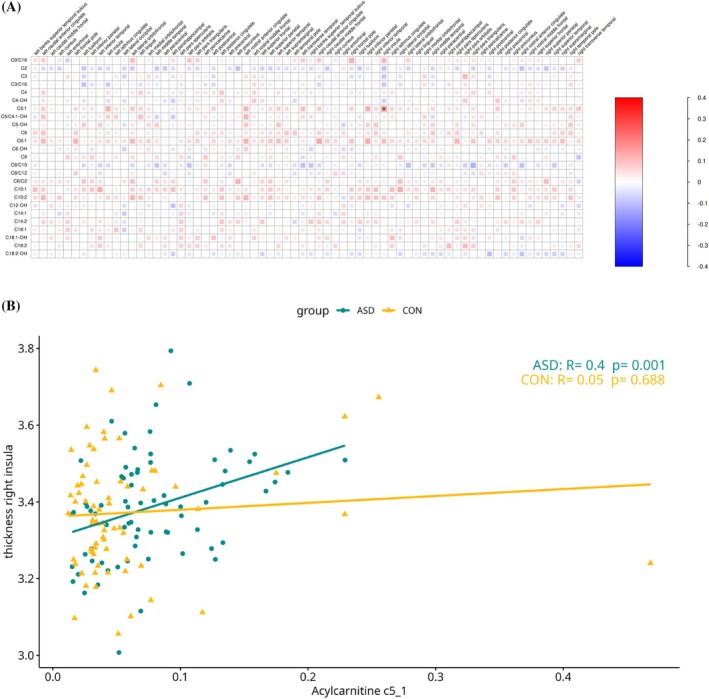
(A) Correlation matrix of acylcarnitine concentration data and cortical thickness across regions of interest in the ASD group. Box color indicates the direction of the correlation, with red indicating a positive correlation and blue indicating a negative correlation. Box size reflects the strength of the correlation, with larger boxes indicating stronger correlations. Significance after correction for multiple testing is indicated by an asterisk (*p* < 0.05). Results showed that C5:1 was positively correlated with cortical thickness in the right insula. (B) Scatterplot showing the association between acylcarnitine C5:1 levels and cortical thickness in the right insula. The yellow line represents the CON group, and the green line represents the ASD group. A significant positive correlation between C5:1 levels and cortical thickness in the right insula was observed within the ASD group. Reported *p*‐values in the plot are shown before correction for multiple testing. Abbreviations: ASD, autism spectrum disorder; CON, control participants; C0/C16, free carnitine/palmitoylcarnitine; C2, acetylcarnitine; C3, propionylcarnitine; C3/C16, propionylcarnitine/palmitoylcarnitine; C4, butyrylcarnitine; C4‐OH, hydroxy‐butyrylcarnitine; C5:1, tiglylcarnitine; C5 + C4:1‐OH, isovalerylcarnitine+3‐hydroxybutyrylcarnitine; C5‐OH, 3‐hydroxy‐isovalerylcarnitine; C6, hexanoylcarnitine; C6:1, hexenoylcarnitine; C6‐OH, 3‐hydroxy‐hexanoylcarnitine; C8, octanoylcarnitine; C8/C10, octanoylcarnitine/decanoylcarnitine; C8/C12, octanoylcarnitine/lauroylcarnitine; C10:1, decanoylcarnitine; C10:2, decadienoylcarnitine; C12‐OH, 3‐hydroxy‐dodecanoylcarnitine; C14:1, tetradecanoylcarnitine; C14:2, tetradecadienoylcarnitine; C18:1, octadecenoylcarnitine; C18:1‐OH, 3‐hydroxy‐oleylcarnitine; C18:2, octadecadienylcarnitine; C18:2‐OH, 3‐hydroxy‐linoleylcarnitine.

Tiglylcarnitine (C5:1) levels showed a significant correlation with CT in the right insula (*r* = 0.40, *p* = 0.001) in the ASD group (Figure 5).

**FIGURE 6:**
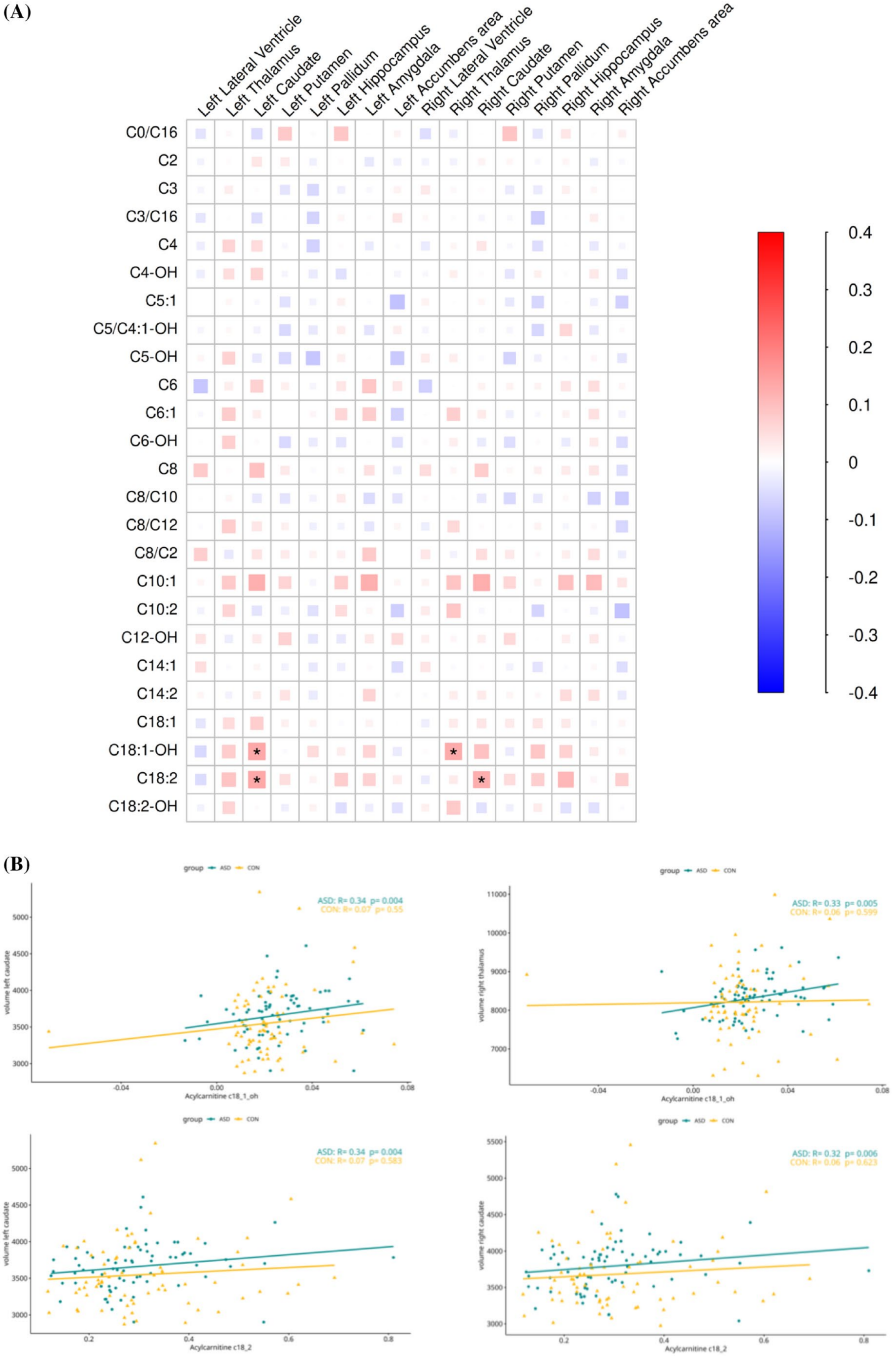
(A) Correlation matrix of acylcarnitine data and subcortical volumes in the ASD group. Box color indicates the direction of the correlation, with red indicating a positive correlation and blue indicating a negative correlation. Box size reflects the strength of the correlation, with larger boxes indicating stronger correlations. Significance after correction for multiple testing is indicated by an asterisk (*p* < 0.05). Results showed that C18:1‐OH was positively correlated with the left caudate nucleus and right thalamus, while C18:2 was positively correlated with both caudate nuclei. (B) Scatterplots showing the associations of acylcarnitine C18:1‐OH with the left caudate nucleus and right thalamus, and of acylcarnitine C18:2 with the left and right caudate nuclei, in the ASD and CON groups. The yellow line represents the CON group, and the green line represents the ASD group. Within the ASD group, significant positive correlations were observed between C18:1‐OH and the left caudate nucleus and right thalamus, as well as between C18:2 and the left and right caudate nuclei. Reported *p*‐values in the plots are shown before correction for multiple testing. Abbreviations: ASD, autism spectrum disorder; CON, control participants; C0/C16, free carnitine/palmitoylcarnitine; C2, acetylcarnitine; C3, propionylcarnitine; C3/C16, propionylcarnitine/palmitoylcarnitine; C4, butyrylcarnitine; C4‐OH, hydroxy‐butyrylcarnitine; C5:1, tiglylcarnitine; C5 + C4:1‐OH, isovalerylcarnitine+3‐hydroxybutyrylcarnitine; C5‐OH, 3‐hydroxy‐isovalerylcarnitine; C6, hexanoylcarnitine; C6:1, hexenoylcarnitine; C6‐OH, 3‐hydroxy‐hexanoylcarnitine; C8, octanoylcarnitine; C8/C10, octanoylcarnitine/decanoylcarnitine; C8/C12, octanoylcarnitine/lauroylcarnitine; C10:1, decanoylcarnitine; C10:2, decadienoylcarnitine; C12‐OH, 3‐hydroxy‐dodecanoylcarnitine; C14:1, tetradecanoylcarnitine; C14:2, tetradecadienoylcarnitine; C18:1, octadecenoylcarnitine; C18:1‐OH, 3‐hydroxy‐oleylcarnitine; C18:2, octadecadienylcarnitine; C18:2‐OH, 3‐hydroxy‐linoleylcarnitine.

3‐hydroxy‐oleylcarnitine (C18:1‐OH) was correlated with SV in the left caudate nucleus (*r* = 0.34, *p* = 0.004) and the right thalamus (*r* = 0.33, *p* = 0.005), while octadecadienylcarnitine (C18:2) was correlated with SV in the left (*r* = 0.34, *p* = 0.004) and right (*r* = 0.32, *p* = 0.006) caudate nuclei in the ASD group (Figure 6A,B).

Posterior‐cingulate Lac^+^ levels were comparable between the ASD and CON groups, and no associations emerged between Lac^+^ and any global or regional morphometric measure after correction for multiple comparisons.

### 3.5 Influence of Psychotropic Medication

After rerunning the significant correlation and between‐group analyses between WURS‐k and CT while adjusting for antipsychotic medication and correcting for multiple testing, the correlation was no longer significant. However, when adjusting for psychostimulants and antidepressants, the pattern of associations remained largely unaltered, with significant correlations observed in multiple frontal, temporal, and parietal cortices. Additionally, a significant negative correlation between CT and EQ was observed in the left frontal pole (*r* = −0.37; *p* = 0.002; see Tables S2–S4, and Figure S1 for full results). Moreover, after adjusting for antidepressants (*p* = 0.013) and psychostimulants (*p* = 0.030), a group difference in the gray matter/TIV ratio reached significance, suggesting these medications may influence global gray matter volume. However, this effect was not observed when adjusting for antipsychotics.

### 3.6 Sensitivity Analysis

In a sensitivity analysis, we repeated the relevant correlation analyses after exclusion of extreme values. The corresponding extreme‐value‐deprived scatter plots are provided in Figures S2–S4.

## 4 Discussion

Consistent with our hypothesis, there were no group differences in GVs. However, when examining specific regions, our hypothesis on subcortical differences was confirmed: the ASD group exhibited enlargement of the caudate nucleus, with no significant cortical differences. Contrary to our hypothesis, no significant correlation was found between ASD symptom severity (SRS‐2, AQ) and brain morphometric traits. However, ADHD symptoms in childhood (WURS‐k) were predictive of widespread reductions in CT.

Correlation analyses between blood metabolites and brain morphometry revealed that acylcarnitine associations were significant. In the ASD group, C3 correlated negatively with TIV; C5:1 correlated positively with CT in the right insula; and two long‐chain acylcarnitines, C18:1‐OH and C18:2, correlated positively with SV (C18:1‐OH with left caudate nucleus and right thalamus; C18:2 with both caudate nuclei). None of these associations were present in the CON group.

### 4.1 Comparison to Previous Studies

In line with previous studies, we found no significant differences in GVs in adults with ASD (Duerden et al. 2012; Mei et al. 2024). Early gray matter and white matter overgrowth in childhood (Courchesne et al. 2001; Herbert et al. 2003) appear to normalize by adulthood (Waiter et al. 2005), suggesting a developmental trajectory shift in ASD brain morphometry (Courchesne et al. 2001; Herbert et al. 2003; Duerden et al. 2012; Lange et al. 2015). Similarly, CT differences tend to diminish over time and often become undetectable in adults (Khundrakpam et al. 2017; van Rooij et al. 2018), which likely explains the absence of significant findings in CT. The interpretation of altered developmental brain trajectories in ASD should also consider the possible influence of childhood symptom severity and therapeutic interventions. Early intervention, timing of diagnosis, and developmental presentation may affect neurodevelopment and contribute to heterogeneity in adult brain morphometry. Because our dataset did not include systematic information on age at diagnosis, childhood severity, or intervention history, we could not examine these factors directly. This should be considered a limitation of the present study and addressed in future longitudinal research. The ENIGMA consortium, however, has reported volume alterations in the amygdala, pallidum, putamen, and nucleus accumbens in ASD (van Rooij et al. 2018; Boedhoe et al. 2020), although subcortical results vary substantially across different studies (Haar et al. 2016; Williams et al. 2020; Weerasekera et al. 2022). For example, caudate nucleus enlargement in individuals with ASD has been repeatedly reported (Langen et al. 2007), and its volume associated with repetitive behaviors (Sears et al. 1999; Hollander et al. 2005; Estes et al. 2011).

While the absence of significant correlations between ASD symptom severity and brain morphometry in our study is in contrast to previous findings (Prigge et al. 2018; Nees et al. 2022), the correlation between ADHD symptoms in childhood and cortical thinning in various regions supports previous findings that ADHD traits are associated with delayed cortical maturation and cortical thinning, particularly in frontal regions involved in executive control and attention regulation (Narr et al. 2009; Shaw et al. 2013; Nickel et al. 2018; Mizuno et al. 2019).

Several studies have reported alterations in metabolic markers in ASD (Narr et al. 2009; Shaw et al. 2013; Nickel et al. 2018; Frye et al. 2019; Kępka et al. 2021).

Thomas et al. (Thomas et al. 2010), using the propionic acid rodent model, demonstrated increased brain acylcarnitine levels, which were associated with ASD‐like behaviors, suggesting that disruptions in fatty acid metabolism and mitochondrial function may contribute to ASD pathophysiology. Beaudet et al. (Beaudet 2017), proposed that brain carnitine deficiency may be a contributing factor, particularly in non‐syndromic autism with an extreme male bias. Frye et al. (Frye et al. 2024) reported that acylcarnitine abnormalities were associated with neurodevelopmental regression and core ASD symptoms, reinforcing the role of disrupted mitochondrial function and fatty acid metabolism in ASD‐related neural and behavioral changes.

Our findings of significant correlations between acylcarnitine levels and regional brain morphometry in ASD suggest that alterations in acylcarnitine metabolism may contribute to ASD pathophysiology.

### 4.2 Significance of Findings

The finding that for numerous brain regions thinner CT was significantly associated with higher childhood ADHD symptom scores in individuals with ASD, while for ASD‐symptom severity no such pattern could be observed, suggests that brain morphometric variance in ASD may potentially be more strongly linked to comorbid ADHD traits rather than core ASD symptoms. Higher ADHD trait burden may index a more complex neurodevelopmental phenotype within ASD. The observed association may reflect shared ASD‐ADHD liability and may also be influenced by increased diagnostic overlap following DSM‐5 that broadened ASD nosology and explicitly allowed comorbid ADHD; therefore, replication in independent ASD samples is warranted. Prior studies have linked ADHD‐related cortical thinning to altered neurodevelopmental trajectories, possibly reflecting disrupted synaptic pruning or delayed maturation (Vaidya 2012; Mous et al. 2014). Reduced CT in individuals with higher ADHD scores may reflect a lower number of neurons within cortical columns in frontal and parietal brain regions, which are crucial for executive function, attention, and impulse control (Shaw et al. 2007; Hoekzema et al. 2012). This underlines the importance of assessing comorbid ADHD in ASD (Leyfer et al. 2006; Hanson et al. 2013). However, after adjusting for antipsychotic medication, these correlations did not remain significant, suggesting that findings may have been influenced by antipsychotic medication effects rather than reflecting a direct neurobiological relationship between ADHD traits and brain morphology. As long‐term antipsychotic use has been shown to impact cortical and subcortical morphology (Feng et al. 2020; Turkheimer et al. 2020; Fan et al. 2023), accounting for psychotropic medication in future studies should be prioritized. Another possible explanation is that the eight ASD participants receiving neuroleptic medication showed significantly higher childhood ADHD symptom scores (see Table S2). As a result, adjusting for medication in the statistical model may have inadvertently removed variance associated with more severe ADHD traits.

Enlargement of the caudate nucleus has been consistently reported in ASD (Hollander et al. 2005; Sato et al. 2014). Repetitive behaviors, a core symptom of autism, are highly prevalent in autistic individuals (Seltzer et al. 2003; Howlin et al. 2004). Several studies have specifically linked caudate volume to repetitive behaviors (Hollander et al. 2005; Voelbel et al. 2006; Langen et al. 2007; Qiu et al. 2016).

Acylcarnitines are esters formed by the conjugation of fatty acids with L‐carnitine, playing a vital role in transporting long‐chain fatty acids into mitochondria for β‐oxidation (Dambrova et al. 2022). Given that altered acylcarnitine levels have been linked to mitochondrial dysfunction, neurodevelopmental regression, and metabolic abnormalities in ASD, their investigation may provide insights into disease mechanisms and potential biomarkers for early diagnosis and targeted interventions (Frye et al. 2013, 2024; Rose et al. 2018; Nickel et al. 2023). Because our analyses are correlational, the present findings cannot establish whether acylcarnitines influence brain structure or merely covary with other, unmeasured factors (see Section 5). The negative correlation between C3 and TIV may point to a relationship between systemic metabolism and global brain development in ASD, although its direction and underlying mechanisms remain to be clarified. The positive association between C5:1 and CT in the right insula, a region involved in sensory integration and social processing, suggests that altered fatty acid metabolism may contribute to atypical cortical development in functionally relevant areas. Finally, the correlations between acylcarnitines (C18:1‐OH; C18:2) and SV, particularly, in the caudate nucleus and thalamus, support a role of fatty acid metabolism in fronto‐striatal circuit alterations. These pathways are critically involved in repetitive behaviors and cognitive flexibility, both of which are important features of ASD.

## 5 Limitations

Despite several strengths, such as the multimodal design combining structural MRI, MRS, the extensive panel of peripheral metabolic biomarkers, and the focus on adults with ASD, a population that remains comparatively understudied, this study has some limitations. The smaller sample size compared to large‐scale multicenter analyses may have constrained the ability to detect small‐to‐moderate effects, particularly, in subgroup analyses; therefore, larger cohorts are needed. Although we adjusted for the use of psychotropic medications, the potential influence of long‐term medication on brain structure and metabolism cannot be entirely ruled out. The absence of a psychiatric control group, such as individuals with ADHD or other neurodevelopmental disorders, limits the specificity of our findings to ASD. In addition, ADHD symptoms were assessed with the WURS‐k, a retrospective self‐report of childhood behavior; reliance on adult recall may introduce bias and misestimate the severity of current ADHD symptoms. Moreover, dietary intake of carnitine may influence metabolic findings, as L‐carnitine is primarily obtained from animal‐based foods. Given that individuals with ASD often exhibit dietary restrictions or preferences, acylcarnitine levels may vary due to nutritional factors rather than intrinsic metabolic dysfunction, suggesting the need for future studies to account for dietary influences (Celestino‐Soper et al. 2012).

## 6 Conclusion

This study contributes to the growing understanding of brain morphometry and metabolic alterations in ASD by examining structural differences in cortical and subcortical regions and their associations with psychometric traits and metabolic measures. In line with previous research, we found that GVs in our adult sample are comparable to those of the CON group, while region‐specific differences, particularly, in the caudate nucleus, remain relevant in adults with ASD. The observed correlations between ADHD symptoms in childhood and CT reinforce the importance of considering co‐occurring trait conditions in ASD research. Additionally, the associations between metabolic biomarkers, including acylcarnitine levels, and brain structures could suggest a potential neurobiological link that warrants further exploration. Future research should focus on longitudinal designs and larger, nutrient‐controlled cohorts to better elucidate the complex interactions between brain morphology, mitochondrial and energy‐metabolism biomarkers, and neurodevelopmental traits in ASD.

## Author Contributions

All authors were critically involved in measurements and/or data analyses, and/or in the theoretical discussion, and/or composition of the manuscript. All authors read and approved the final version of the manuscript.

## Funding

The study was funded by the “German Research Foundation” (DFG ID: MA 7813/1‐1, TE 280/15‐1).

## Conflicts of Interest

K.D.: Member of the Neurotorium Editorial Board, The Lundbeck Foundation. L.T.v.E.: Advisory boards, lectures, or travel grants within the last 3 years: Roche, Eli Lilly, Janssen‐Cilag, Novartis, Shire, UCB, GSK, Servier, Janssen, and Cyberonics. The other authors declare no conflicts of interest.

## Supporting information


**Table S1:** Structural quality measures.
**Table S2:** Psychometric measures in relation to antipsychotic medication use.
**Table S3:** Cortical thickness versus WURS‐K after adjusting for antidepressant use.
**Table S4:** Cortical thickness versus WURS‐K after adjusting for psychostimulant use.
**Figure S1:** The figure illustrates the influence of medication use on the relationship between psychometric traits and cortical thickness in individuals with ASD.
**Figure S2:** Scatterplot showing the association between propionylcarnitine C3 levels and TIV in the ASD and CON groups after exclusion of extreme values in a sensitivity analysis.
**Figure S3:** Scatterplot showing the association between acylcarnitine C5:1 levels and cortical thickness in the right insula, after exclusion of extreme values in a sensitivity analysis.
**Figure S4:** Scatterplots showing the associations of acylcarnitine C18:1‐OH with the left caudate nucleus and right thalamus and of acylcarnitine C18:2 with the left and right caudate nuclei, in the ASD and CON groups after exclusion of extreme values in a sensitivity analysis.

## Data Availability

The data that support the findings of this study are available on request from the corresponding author. The data are not publicly available due to privacy or ethical restrictions.
