# Supplementary material for: Linking Brain Morphometry to Psychometric Measures and Energy‐Metabolic Biomarkers in Adults With Autism Spectrum Disorder
Source: Autism Res. 2026 Jun 5;19(7):e70288. doi: 10.1002/aur.70288 (PMC13377343; doi:10.1002/aur.70288)
Supplement: Supplementary file 1 — Table S1: Structural quality measures. Table S2: Psychometric measures in relation to antipsychotic medication use. Table S3: Cortical thickness versus WURS‐K after adjusting for antidepressant use. Table S4: Cortical thickness versus WURS‐K after adjusting for psychostimulant use. Figure S1: The figure illustrates the influence of medication use on the relationship between psychometric traits and cortical thickness in individuals with ASD. Figure S2: Scatterplot showing the association between propionylcarnitine C3 levels and TIV in the ASD and CON groups after exclusion of extreme values in a sensitivity analysis. Figure S3: Scatterplot showing the association between acylcarnitine C5:1 levels and cortical thickness in the right insula, after exclusion of extreme values in a sensitivity analysis. Figure S4: Scatterplots showing the associations of acylcarnitine C18:1‐OH with the left caudate nucleus and right thalamus and of acylcarnitine C18:2 with the left and right caudate nuclei, in the ASD and CON groups after exclusion of extreme values in a sensitivity analysis. [file AUR-19-0-s001.docx]

**SUPPLEMENTARY MATERIAL**

| **Supplementary Table 1.** **Structural quality measures** | | | |
| --- | --- | --- | --- |
| Characteristic | ASD  N = 74^1^ | CON  N = 71^1^ | p-value^2^ |
| CAT12 image quality rating | 1.836 ± 0.004 | 1.837 ± 0.007 | 0.20 |
| Noise/contrast ratio | 0.91 ± 0.24 | 0.98 ± 0.25 | 0.084 |
| Surface defect number | 1.59 ± 0.24 | 1.64 ± 0.32 | 0.30 |
| Surface defect area | 1.11 ± 0.07 | 1.14 ± 0.12 | 0.12 |
| ^1^Mean ± SD | | | |
| ^2^Welch Two Sample t-test | | | |
| Abbreviations: ASD, autism spectrum disorder; CON, control participants. | | | |

No significant group differences were observed in structural image quality measures, including the CAT12 image quality rating, noise-to-contrast ratio, number of surface defects, and surface defect area.

| **Supplementary Table 2.** Psychometric measures in relation to antipsychotic medication use | | | |
| --- | --- | --- | --- |
| Characteristic | No Antipsychotics  N = 66^1^ | Antipsychotics  N = 8^1^ | p-value^2^ |
| AQ | 35.7 ± 8.1 | 35.1 ± 8.4 | 0.80 |
| EQ | 21.9 ± 11.1 | 23.6 ± 11.6 | 0.60 |
| SRS2 | 105.5 ± 28.1 | 130.4 ± 25.8 | 0.014 |
| WURS-k | 26.3 ± 15.5 | 41.3 ± 15.7 | 0.032 |
| BDI-II | 14.5 ± 12.2 | 25.8 ± 16.4 | 0.06 |
| ^1^Mean ± SD | | | |
| ^2^Wilcoxon rank sum test | | | |
| Abbreviations: AQ, Autism Spectrum Quotient; BDI-II, Beck Depression Inventory-II; EQ, Empathy Quotient; SD, Standard Deviation; SRS-2, Social Responsiveness Scale-2; WURS-k, Wender Utah Rating Scale. | | | |

The table shows psychometric measures in relation to antipsychotic medication use. Data are presented as mean ± standard deviation. Group differences were assessed using the Wilcoxon rank sum test. Significant differences were observed for SRS-2 and WURS-k scores, with higher scores among individuals taking antipsychotic medication.

| **Supplementary Table 3.** Cortical thickness vs WURS-K after adjusting for antidepressant use | | | |
| --- | --- | --- | --- |
| **Hemisphere** | **Region** | ***r*** | ***p-value*** |
| Left | Frontal pole | -0.30 | 0.024 |
| Left | Caudal middle frontal gyrus | -0.32 | 0.008 |
| Left | Insula | -0.31 | 0.011 |
| Left | Middle temporal gyrus | -0.32 | 0.007 |
| Left | Pars opercularis | -0.40 | 0.001 |
| Left | Pars orbitalis | -0.30 | 0.012 |
| Left | Pars triangularis | -0.37 | 0.002 |
| Left | Posterior cingulate cortex | -0.34 | 0.004 |
| Left | Precentral gyrus | -0.37 | 0.002 |
| Left | Precuneus | -0.32 | 0.007 |
| Left | Superior frontal gyrus | -0.31 | 0.009 |
| Right | Caudal middle frontal gyrus | -0.38 | 0.001 |
| Right | Inferior parietal lobule | -0.30 | 0.011 |
| Right | Pars opercularis | -0.38 | 0.001 |
| Right | Pars triangularis | -0.30 | 0.006 |
| Right | Precentral gyrus | -0.35 | 0.004 |
| Right | Supramarginal gyrus | -0.35 | 0.003 |

The table presents correlations between cortical thickness and WURS-K scores, after adjusting for antidepressant use. Analyses were conducted separately for the left and right hemispheres. Only regions with *p* < 0.05 are shown. Negative *r* values indicate that higher WURS-K scores (reflecting greater ADHD-related symptoms) are associated with reduced cortical thickness in the corresponding region.

| **Supplementary Table 4.** Cortical thickness vs WURS-K after adjusting for psychostimulant use | | | |
| --- | --- | --- | --- |
| **Hemisphere** | **Region** | ***r*** | ***p-value*** |
| Left | Frontal pole | -0.29 | 0.017 |
| Left | Caudal middle frontal gyrus | -0.36 | 0.003 |
| Left | Lateral orbitofrontal cortex | -0.31 | 0.009 |
| Left | Middle temporal gyrus | -0.33 | 0.006 |
| Left | Pars opercularis | -0.38 | 0.001 |
| Left | Pars orbitalis | -0.32 | 0.006 |
| Left | Pars triangularis | -0.37 | 0.002 |
| Left | Posterior cingulate cortex | -0.33 | 0.006 |
| Left | Precentral gyrus | -0.36 | 0.002 |
| Left | Precuneus | -0.33 | 0.006 |
| Left | Superior frontal gyrus | -0.32 | 0.008 |
| Right | Caudal middle frontal gyrus | -0.39 | 0.001 |
| Right | Inferior parietal lobule | -0.33 | 0.006 |
| Right | Pars opercularis | -0.38 | 0.001 |
| Right | Precentral gyrus | -0.32 | 0.008 |
| Right | Supramarginal gyrus | -0.35 | 0.003 |

The table presents correlations between cortical thickness and WURS-K scores, after adjusting for psychostimulant use. Analyses were conducted separately for the left and right hemispheres. Only regions with *p* < 0.05 are shown. Negative *r* values indicate that higher WURS-K scores (reflecting greater ADHD-related symptoms) are associated with reduced cortical thickness in the corresponding region.

|  |
| --- |

**Supplementary Figure 1.** The figure illustrates the influence of medication use on the relationship between psychometric traits and cortical thickness in individuals with ASD. Box color represents the direction of the correlation, with green indicating a positive correlation and purple indicating a negative correlation. Box size reflects the strength of the correlation, with larger boxes corresponding to stronger associations. Asterisks denote statistical significance (p < 0.05). Each panel shows partial correlations under different adjustment conditions.

Abbreviations: ASD, autism spectrum disorder.

| 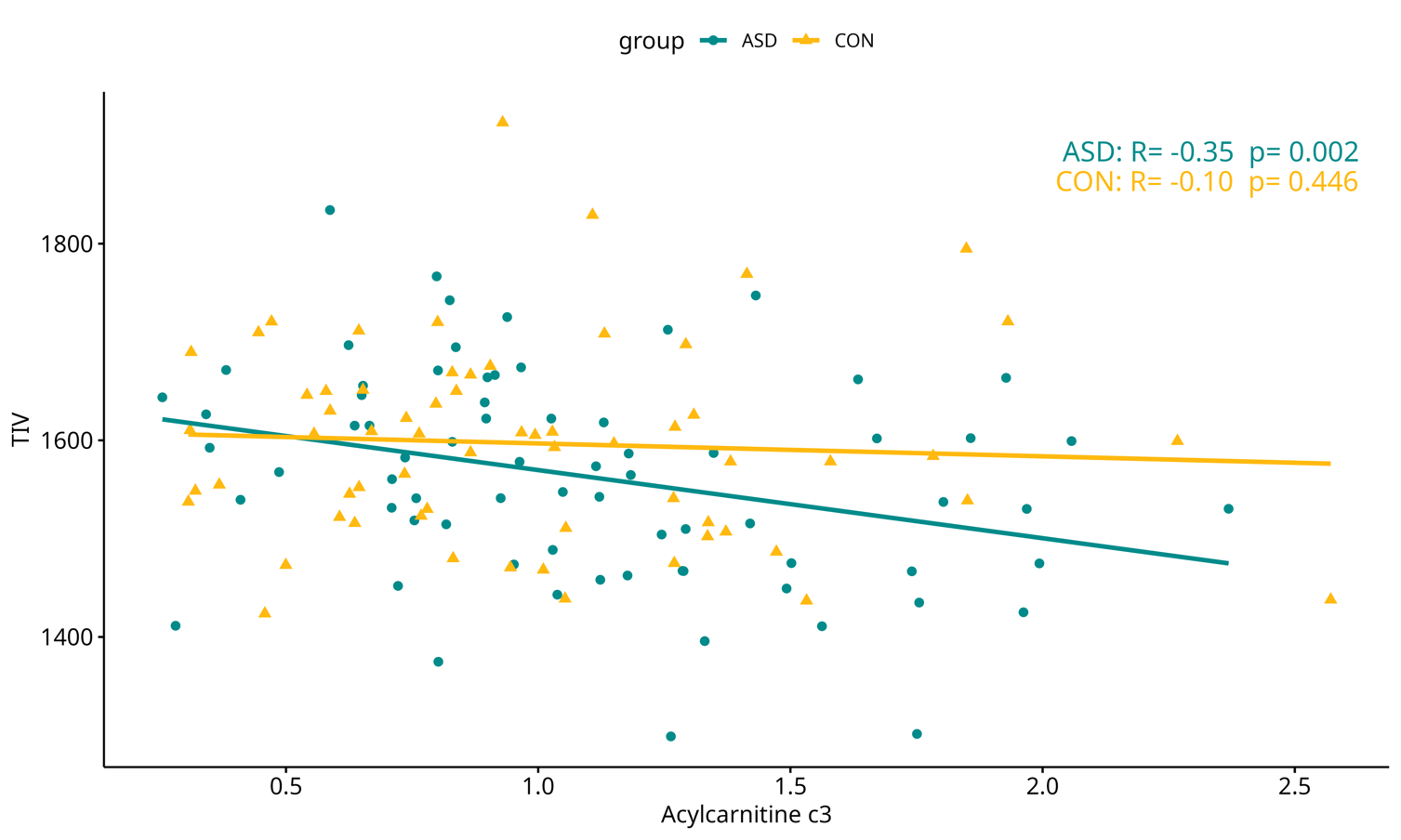 |
| --- |

**Supplementary Figure 2.** Scatterplot showing the association between propionylcarnitine C3 levels and TIV in the ASD and CON groups after exclusion of extreme values in a sensitivity analysis. Extreme values were identified separately within each diagnostic group using modified z-scores based on the median absolute deviation (MAD), applying a threshold of |modified z| > 3.5 on either axis. In this analysis, 6 observations were excluded in total (1 ASD, 5 CON). The yellow line represents the CON group, and the green line represents the ASD group. A significant negative correlation between C3 and TIV was observed in the ASD group after correction for multiple testing. Reported *p-*values shown in the plot are uncorrected. Abbreviations: ASD, autism spectrum disorder; CON, control participants; C3, propionylcarnitine; TIV, total intracranial volume.

| 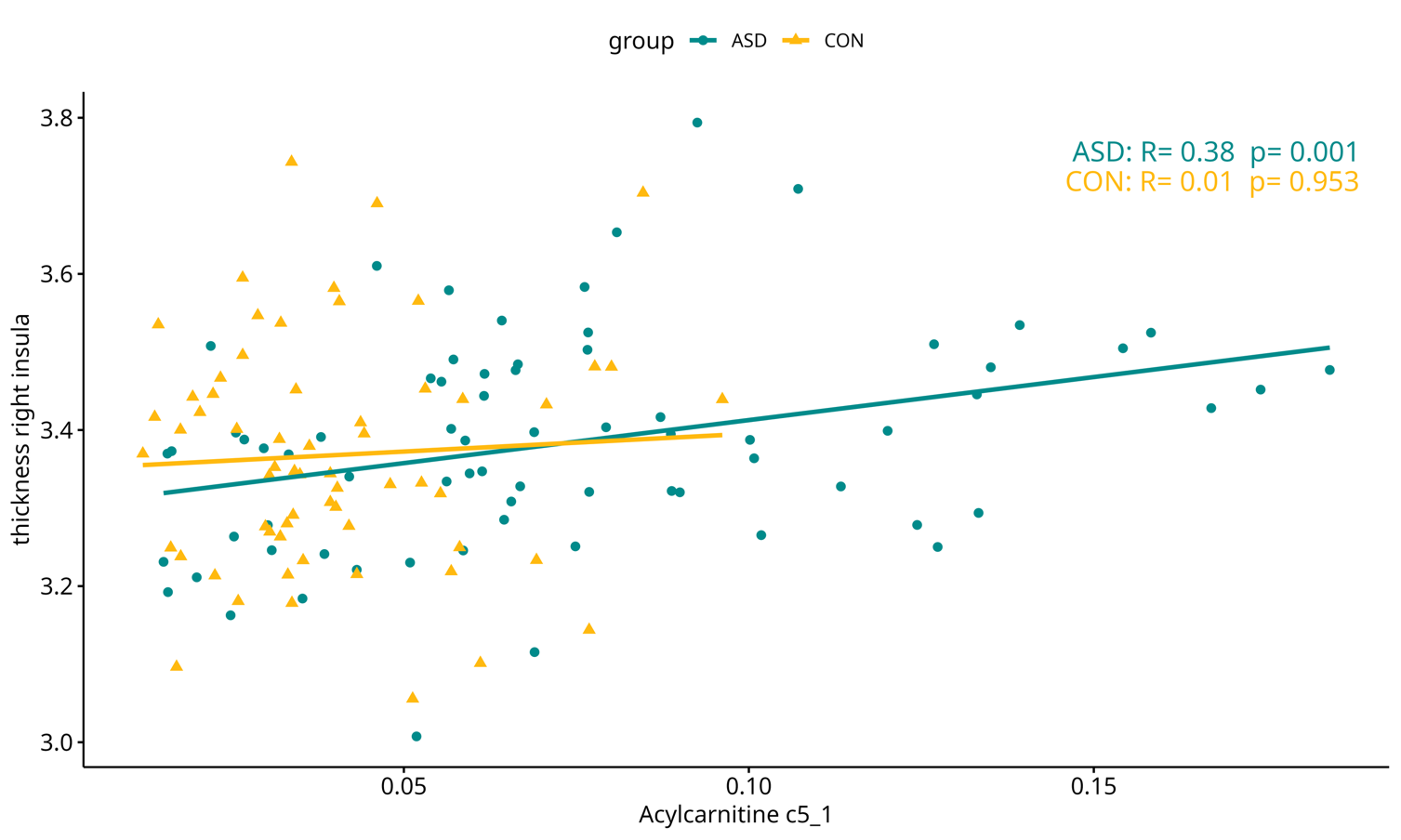 |
| --- |

**Supplementary Figure 3.** Scatterplot showing the association between acylcarnitine C5:1 levels and cortical thickness in the right insula, after exclusion of extreme values in a sensitivity analysis. Extreme values were identified separately within each diagnostic group using modified z-scores based on the median absolute deviation (MAD), applying a threshold of |modified z| > 3.5 on either axis. In this analysis, 8 observations were excluded in total (1 ASD, 7 CON). The yellow line represents the CON group, and the green line represents the ASD group. A significant positive correlation between C5:1 and CT in the right insula was observed in the ASD group after correction for multiple testing. Reported *p-*values shown in the plot are uncorrected. Abbreviations: ASD, autism spectrum disorder; CON, control participants; C5:1, tiglylcarnitine.

|  |
| --- |

**Supplementary Figure 4.** Scatterplots showing the associations of acylcarnitine C18:1-OH with the left caudate nucleus and right thalamus and of acylcarnitine C18:2 with the left and right caudate nuclei, in the ASD and CON groups after exclusion of extreme values in a sensitivity analysis. Extreme values were identified separately within each diagnostic group for each scatterplot using modified z-scores based on the median absolute deviation (MAD), applying a threshold of |modified z| > 3.5 on either axis. The yellow line represents the CON group, and the green line represents the ASD group. Within the ASD group, significant positive correlations were observed between C18:1-OH and the left caudate volume and right thalamic volume, and between C18:2 and left and right caudate volumes. Reported *p*-values shown in the plots are uncorrected. The number of excluded observations for each panel was as follows: C18:2-left caudate, n = 8 (4 ASD, 4 CON); C18:2-right caudate, n = 8 (4 ASD, 4 CON); C18:1-OH-left caudate, n = 7 (0 ASD, 7 CON); and C18:1-OH-right thalamus, n = 6 (0 ASD, 6 CON). Abbreviations: ASD, autism spectrum disorder; CON, control participants; C18:1-OH, 3-hydroxy-oleylcarnitine; C18:2, octadecadienylcarnitine.
